# Green Synthesis, Characterization, Antimicrobial, Anti-Cancer, and Optimization of Colorimetric Sensing of Hydrogen Peroxide of Algae Extract Capped Silver Nanoparticles

**DOI:** 10.3390/nano10091861

**Published:** 2020-09-17

**Authors:** Abdelaziz Elgamouz, Hamid Idriss, Chahlaa Nassab, Alaa Bihi, Khalid Bajou, Kamrul Hasan, Mohammad Abu Haija, Shashikant P. Patole

**Affiliations:** 1 Department of Chemistry, College of Sciences, University of Sharjah, Sharjah PO. Box 27272, UAE; hdrisom@sharjah.ac.ae (H.I.); U19103696@sharjah.ac.ae (C.N.); U14220224@sharjah.ac.ae (A.B.); khasan@sharjah.ac.ae (K.H.); 2 Department of Applied Biology, College of Sciences, University of Sharjah, Sharjah PO. Box 27272, UAE; kbajou@sharjah.ac.ae; 3 Department of Chemistry, Khalifa University of Science and Technology, Abu Dhabi PO. Box 127788, UAE; mohammad.abuhaija@ku.ac.ae; 4 Department of Physics, Khalifa University of Science and Technology, Abu Dhabi PO. Box 127788, UAE; shashikant.patole@ku.ac.ae

**Keywords:** silver nanoparticles, hydrogen peroxide, biosensor, cancer cells, bacterial inhibition

## Abstract

A green and cost-effective technique for the preparation of silver nanoparticles (Algae-AgNPs) as a colorimetric sensor for hydrogen peroxide (H_2_O_2_) is described. Silver nanoparticles were capped using the green algae *(Noctiluca scintillans*) extract at an optimum time of 3 h at 80 °C. The pH of the plant extract (pH = 7.0) yields nanoparticles with a mean size of 4.13 nm and a zeta potential of 0.200 ± 0.02 mV and negative polarity, using dynamic light scattering (DLS). High-resolution transmission electron microscopy (HRTEM) analysis showed regular spherical particles with the average size of 4.5 nm. Selected area electron diffraction (SAED) results revealed the polycrystalline nature of the silver nanoparticles. The obtained patterns were indexed as (111), (200), (220), and (311) reflections of the fcc (face centered cubic) silver crystal based on their d-spacing of 2.47, 2.13, 1.49, and 1.27 Å, respectively. The apparent color change from brown to colorless was observed when nanoparticles reacted with H_2_O_2_. Linear responses were obtained in three different ranges (nM, µM, and mM). Limits of detection (LOD) of 1.33 ± 0.02 and 1.77 ± 0.02 nM and quantitation limits (LOQ) of 7.31 ± 0.03 and 9.67 ± 0.03 nM were obtained for Abs and ΔAbs calibration curves, respectively. 10% *v*/*v* Algae-AgNPs solution inhibited *Staphylococcus aureus* over *Escherichia coli*, while a 50% reduction of tumor cell growth of MDA-MB-231 human breast adenocarcinoma was obtained.

## 1. Introduction

Hydrogen peroxide (H_2_O_2_) is one of the few chemicals that are available to the public for sale in stores. It is used as an antiseptic and is indicated for small dermal scratches; in mouth, gum, teeth pain, or whitening and oral discharge [1]. H_2_O_2_ has multiple uses such as the disinfection of waters because of its association with in situ chemical reactions [2] and its ability to destroy a wide spectrum of bacteria, including molds, yeasts, viruses, and spore-forming organisms [3]. H_2_O_2_ is reasonably safe to manipulate and it is preservative-free, which makes it more suitable for people allergic to preservatives found in multipurpose solutions [4]. Ten percent *v*/*v* solutions of H_2_O_2_ are considered dangerous, must not be ingested, and can cause systematic toxicity. Solutions below 9% are considered nontoxic; however, 3% solutions can be mildly irritating to mucosal tissue and may cause vomiting and diarrhea [5].

H_2_O_2_ was once widely used as a food preservative because of its biopreservation character to microorganisms. H_2_O_2_ is produced through these microorganisms’ metabolites; for example, probiotic lactic acid bacterium Lactobacillus johnsonii NCC 533 excretes up to 1 mM H_2_O_2_ when it is exposed to oxygen, leading to cell death [6]. H_2_O_2_ and other antimicrobial substances (organic acids, diacetyl, acetoin) could inhibit foodborne pathogens and decompose microorganisms [7]. At low concentrations, H_2_O_2_ plays a clinical role as a signaling mediator of acute diseases [8]. H_2_O_2_ subsequently is present with considerable amounts in saliva and breast milk and works as a substrate for the milk enzyme lactoperoxidase (LPO) to convert dietary substances into antibacterial reactive oxygen species [9]. Breast milk, especially colostrum, contains very high concentrations of H_2_O_2_. One of the main functions of breast milk is to boost the infant’s intake from free radicals with the aim of activating and stimulating the infant’s immune system to be competent [10]. The H_2_O_2_ stable form in human blood is balancing between a lower concentration of 0.25 μM, a possible lower range of 1–5 μM, and a possible high range of 30–50 μM, which is usually found during some diseases or chronic inflammation [11]. Alzheimer’s disease and Parkinson’s disease are examples when H_2_O_2_ could be one of the main causes of disease development and progression [12].

In the early nineteenth century, H_2_O_2_ was widely used for the treatment of many diseases, including syphilis, whooping cough, cholera, typhoid fever, ulcers, and cancer. This kind of therapy is called oxidative therapy, which is based on flooding the cells with oxygen from an oxygenated compound, such as H_2_O_2_, which apparently kills the vulnerable cell by supplying more oxygen than what it could sustain [13]. Later, no evidence was found showing that H_2_O_2_ is safe or results in objective benefit in the treatment of cancer. Hence, the US Food and Drug Administration (FDA) had warned consumers against using high concentrations of H_2_O_2_ solutions for medical purposes due to its ability to cause serious harm and possibly lead to death [14].

The role of H_2_O_2_ in physiology and pathophysiology is not well understood. Hereafter, researchers are developing various analytical methods, including in vivo, in vitro, and ex vivo techniques, to quantify the level of H_2_O_2_. Light-detecting sensors and electrochemical methods are the most advantageous among many other techniques used for H_2_O_2_ detection. Light detection sensors usually alter the structure of H_2_O_2_ through the absorption or emission of light from a chemical substance.

Metal nanoparticles have been widely used as biosensors, as tracers of MRI, in cell targeting therapy, and in catalysis due to unique properties that do not exist in their parent bulk materials [15,16]. Especially, the localized surface plasmon resonance (LSPR) is responsible for the colors or emissions of nanoparticles that can be detected in the UV-visible region using either the Abs or emission of samples.

Silver nanoparticles (AgNPs) have had special scientific focus because they exhibit a wide spectrum of bacterial and fungicidal activity [17,18,19]. In addition, AgNPs were found to be advantageous over other noble metal nanoparticles such as gold nanoparticles (AuNPs) because of their important physicochemical properties, low cost, thermal and electrical conductivity, and more importantly, the small loss of optical frequency during the surface plasmon propagation [20]. Silver nanoparticles were used in many industries such as antiseptic sprays, wound dressing, and topical creams because of their antibacterial character [21]. The mechanism of action of AgNPs is based on the association of the nanoparticles shell to the bacterial cell wall, which results in alteration of the bacterial cell membrane and subsequently cell death [22]. On the contrary, gold nanoparticles’ (AuNPs) antibacterial activities are dependent on the shape and size of the nanoparticles. However, the fundamental antimicrobial mechanism of AuNPs is similar to AgNPs in that the capping shells of AuNPs attach to bacterial cell membranes and disrupt its structure, leading to the increase of permeability and inhibition of the respiratory function of the bacterial cell and finally death [23,24].

Several approaches were used for the synthesis of AgNPs, mostly those using hazardous synthetic reducing agents that possess subchronic and chronic toxicity [25]. Consequently, plant extracts could be used as reducing and shell-stabilizing agents to form stable AgNPs using a green chemistry approach as an alternative method over other toxic chemical reducing agents [26,27,28]. Plants extracts are rich with plant secondary metabolite phytochemicals with various functional groups that can ease the reduction and stabilization of AgNPs [29,30]. The use of plant extract as a reducing/capping shell is advantageous because these molecules could cross the cell membranes by specific recognition through mediators. In addition to their sustainability and environmentally friendly character, they are cost-effective. Moreover, the use of plant extracts as capping agents in AgNPs leads to the formation of small size nanoparticles through slow kinetic reactions, avoiding the formation of aggregates. For example, AgNPs were synthesized using biocompatible materials such as biopolymers [31,32,33,34,35], natural aluminosilicates [36], and plant extracts [37,38,39,40]. Herein, an environmentally friendly method for the synthesis of sea-green algae extract stabilized AgNPs is described. Their use as a colorimetric sensor for H_2_O_2_ and their bacterial and anti-cancer proliferation activities were investigated. Green algae was used instead of terrestrial plants because of its higher biological activity and availability. In addition, algae grows much faster than terrestrial plants; besides that, oceans account for more than 70% of the Earth’s surface, which makes them a large reservoir of natural resources [41,42].

## 2. Experimental Part

### 2.1. Algae Collection and Plant Extract Preparation

*Noctiluca scintillans is* a common algal species found on the Oman Gulf in the eastern coastal sea of the United Arab Emirates. These species are not harmful in small amounts but must be monitored, as higher concentrations could pose environmental threats. Algae were collected from the top of rocks, washed six times with distilled water, and dried in open air at a temperature between 32 and 36 °C. Dried algae sample was ground in a mortar; then, 2.0 g of the dried sample were suspended in 100 mL of deionized water, stirred vigorously for 2 h, and boiled for 30 min. The extract was filtered using a 1.0 µm Whatman filter for 12 h and stored in the fridge for further utilization.

### 2.2. Materials and Reagents

All chemicals were used as received, unless otherwise stated. D-(+)-Glucose (C_6_H_12_O_6_, ≥99.5% (GC)), urea (grade for analysis ACS, reagent), hydrogen peroxide (H_2_O_2_, 35% *w*/*w*, d = 1.13 g mL^−1^), buffer pH = 4.0 (citric acid/sodium hydroxide), buffer pH = 7.0 (potassium dihydrogen phosphate, disodium hydrogen phosphate, 12 hydrates), buffer pH = 10.0 (borax/sodium hydrate), sodium nitrate (NaNO_3_, ≥99.0%), ammonium nitrate (NH_4_ NO_3_, ACS reagent, ≥98%), potassium chloride (KCl, for analysis EMSURE), sodium dihydrogen phosphate (NaH_2_PO_4_, anhydrous 99.99% Suprapur), calcium nitrate (Ca(NO_3_)_2_, ≥99.0%), Cobalt(II) nitrate hexahydrate (Co(NO_3_)_2_ ·6H_2_O, ACS reagent), nickel(II) nitrate hexahydrate (Ni(NO_3_)_2_·6H_2_O, ≥98.0%), copper(II) nitrate hydrate (Cu(NO_3_)_2_·2.5H_2_O, ACS reagent, 98%), zinc nitrate hexahydrate (Zn(NO_3_)_2_·6H_2_O, reagent grade, 98%), and lead(II) nitrate (Pb(NO_3_)_2_, ACS reagent, ≥99.0%) were purchased from Sigma Aldrich. Silver nitrate (AgNO_3_, ≥99.9%) was purchased from Alfa Aesar. Agar powder, bacteriological grade was purchased from Hemedia. Dulbecco’s Modified Eagle’s Medium (DMEM) supplemented with 10% *v*/*v* fetal bovine serum (FBS), 100 μg/mL penicillin, 100 μg/mL streptomycin, MTT (3-(4, 5- thiazol-2yl)-2, 5-diphenyl tetrazolium bromide), and dimethyl sulfoxide (DMSO, (CH_3_)_2_SO, ≥99%, food grade). MilliQ ultra-pure water (conductance = 17.5 MΩ) was used to prepare aqueous solutions.

### 2.3. Instrumentation

The morphology, distribution, and composition of the capped nanoparticles were visualized and analyzed using a Tescan VEGA XM (Tescan, Brno, Czech Republic) variable pressure SEM equipped with an Oxford Instruments X-Max 50 EDS detector, controlled with AZtecEnergy analysis software with a resolution of 125 eV to determine the abundance of elements. SEM images were taken at an accelerating voltage of 30 kV controlled by VEGA TC software. The Algae-AgNPs were transferred onto the TEM grid (holey carbon on a copper grid with 300 mesh from Electron Microscopy Sciences) for further analysis. The structural characterization was performed by employing an aberration-corrected TEM (Hillsboro, Oregon, USA, Thermo Fisher Scientific formerly FEI, Titan G2) at 300 kV equipped with a Cs probe corrector. The particles’ size distribution was determined using a dynamic light scattering Nanotrac Wave II DLS analyzer (Microtrac, Pennsylvania, USA) in the zeta potential mode and operating between 0.8 nm and 6.5 μm. Results were obtained after 15 cycles at a 90° angle and 26 °C. UV-vis absorbances were measured using a Thermo Scientific Multiskan 1510-02828C spectrophotometer. It was used in spectral scanning, endpoint, and kinetic measurements of Abs in the 200–1000 nm wavelength ranges from appropriate 96-well plates and cuvettes. The final concentrations of H_2_O_2_ were evaluated from the calibration curves of Algae-AgNPs reacted with different concentrations of H_2_O_2_ in three different ranges: nM, µM, and mM. The Abs of Algae-AgNPs and ΔAbs, which represents the difference between reference Algae-AgNPs and the Abs of H_2_O_2_ reacted with Algae-AgNPs, were used to assess the level of H_2_O_2_. The development of purple color visualized the presence of viable cells for the anticancer activity, and optical density was read using the 96-well plates at 590 nm. Incubations for the antibacterial and anticancer cell proliferation were carried out at 37 °C in a Shel Lab 2300 incubator (Sheldon Manufacturing Incorporated, Oregon, USA). All experiments were carried out at 25 °C, except when the effect of temperature was studied. The initial pH values of the solutions containing H_2_O_2_ were recorded without adjustment in the experiment of Algae-AgNPs mass effect; the pH was adjusted to 7.0 in all other experiments and to the desired pH when the effect of pH was studied. Abs at 436 nm was read against deionized water or buffer when the pH effect was studied. The limit of detection (LOD) of the Algae-AgNPs test was determined from the standard deviation of the response and slope of the calibration curve following the equation LOD = 3.3 σ/S [43], where σ is the standard deviation of 10 replicates of the lowest concentration, made by mixing 0.75 mL of Algae-AgNPs with 100 µL of H_2_O_2_ of lowest concentration to give a total volume of 0.95 mL, and Abs was read at 436 nm. The limit of quantitation (LOQ) was determined from the equation LOQ = 10 L_v_/S, where L_v_ is the lowest concentration detected by the test. S represents the slope of the calibration curve in the nM or µM ranges. All results were calculated from the averages of at least three sample readings and represented as mean ± SD.

### 2.4. Synthesis of Algae Extract Stabilized Silver Nanoparticles

Algae-capped silver nanoparticles were synthesized using 2.0% *m*/*v* algae extract and 0.1 M AgNO_3_. The synthesis of the nanoparticles and detection of H_2_O_2_ were optimized by studying the AgNO_3_ concentration/plant extract ratios, time, pH medium, and temperature effects on the formation of nanoparticles. The optimization of H_2_O_2_ was carried out by studying the effect of the Algae-AgNPs amount, time of contact, pH, and temperature of the H_2_O_2_ solutions. Batch experiments’ methods were used to find the optimum conditions.

### 2.5. Effect of AgNO_3_ Concentration and Plant Extract Ratio (Plant Extract/AgNO_3_) on the Formation of Nanoparticles and the Effect of Algae-AgNPs Volume on the Detection of H_2_O_2_

In amber bottles, 10 mL of 2.0% *m*/*v* algae extract and 0.1 M AgNO_3_ were mixed in ratios ranging from 1:9 to 9:1 *v*/*v* AgNO_3_/plant extract. The solutions were vigorously stirred and heated to 80 °C for 5 h. The effect of the amount of Algae-AgNPs on H_2_O_2_ detection was studied by adding fixed volumes of 0.75 mL from 40 mM H_2_O_2_ solution to a cuvette containing volumes of Algae-AgNPs ranging from 0.75 to 2.25 mL; each solution was calibrated at 25 °C in the cuvette prior to UV-Vis analysis. The experiments were performed without pH adjustments.

### 2.6. Effect of Time on the Preparation of Algae-AgNPs and on the Detection of H_2_O_2_ at 25 °C

From the previous experiment, the optimum ratio of AgNO_3_/plant extract was found to be 3:7 *v*/*v*. This ratio was used to study the kinetic of Algae-AgNPs formation. In an amber bottle, 3.0 mL of 0.1 M AgNO_3_ was mixed with 7.0 mL of plant extract. Aliquots of 300 µL were collected every hour and analyzed using the microplate reader at a wavelength of 436 nm. However, to evaluate the time required to attain equilibrium for H_2_O_2_ detection using the Algae-AgNPs, 0.75 mL of 40 mM H_2_O_2_ solution was mixed with 0.75 mL of Algae-AgNPs in the cuvette, and a kinetic loop was set to take readings every 30 s at contact times ranging from 0.5 to 30 min at 25 °C on the spectrophotometer.

### 2.7. Effect of pH on the Preparation of Algae-AgNPs and Detection of H_2_O_2_ at 25 °C

The effect of pH was studied through the kinetic of the Algae-AgNPs at four different pH values: 2.4, 4.10, 7.2 (pH of the plant extract), and 10.2. Acidic pH was adjusted using 0.1 M HCl solution, while basic pH was adjusted using 3.0 M ammonia. Samples were analyzed in the same way as the kinetic experiments; every hour, 300 µL aliquots from each pH batch were collected and analyzed using the microplate reader. The pH effect on H_2_O_2_ detection was studied by adjusting the pH of H_2_O_2_ stock solutions to pH = 4.0, 7.0, and 10.0 using buffer solutions. Then, 0.75 mL of 40.0 mM H_2_O_2_ was added to cuvettes containing 0.75 mL Algae-AgNPs for each pH. Kinetic loops were set to take readings every 30 s at contact times ranging from 0.5 to 30 min at 25 °C.

### 2.8. Effect of the Temperature on the Preparation of Algae-AgNPs and H_2_O_2_ Detection

The effect of temperature was studied at three different temperatures: 25, 80, and 90 °C. In three amber bottles, the optimum ratio of AgNO_3_/plant extract (3.0 mL of 0.1M AgNO_3_/7.0 mL of plant extract) were mixed; then, they were kept at the respective temperature for 5 h under vigorous stirring of 700 rpm. The Abs of 300 µL aliquot was measured every hour. The temperature effect on H_2_O_2_ detection was studied by adjusting the temperature of the spectrophotometer to 25, 37, and 40 °C prior to analysis. Then, 0.75 mL of 40.0 mM H_2_O_2_ was added to cuvettes containing 0.75 mL Algae-AgNPs for each specific temperature. Kinetic loops were set to take readings every 30 s at contact times ranging from 0.5 to 30 min.

### 2.9. Calibration Curves and Unknown Analysis

Calibration curves for the detection of H_2_O_2_ using Algae-AgNPs were made in three different ranges: 4.7 to 32 nM; 4.7 to 32 µM; and 4.7 to 32 mM. Volumes of H_2_O_2_ from 40 nM, 40 µM, and 40 mM stock solutions were varied between 0.1 and 2.8 mL with a step increase of 0.1 mL and mixed with 0.75 mL of Algae-AgNPs. Unknown H_2_O_2_ solutions were taken as 0.3 mL H_2_O_2_ solution from each stock solution, which should theoretically give final concentrations of 11.4 nM, 11.4 µM, and 11.4 mM for the three ranges, respectively. Abs values of 0.75 mL of the three solutions were recorded after mixing with 0.75 mL of Algae-AgNPs. The experiments were performed 10 times, and the concentrations of unknown samples were determined from the calibration curves.

### 2.10. Disk Diffusion Test

The inhibition capacity of the Algae-AgNPs was tested on two human pathogenic bacteria, *Escherichia coli* ATCC25922 and *Staphylococcus aureus* ATCC29213 strains (American Type Culture Collection). Both strains were grown in Luria–Bertani (LB) media and plated on nutrient agar for the diffusion test. Bacterial inhibition growth was determined by measuring the size of the inhibition zone in agar plates. Nutrient agar plates were prepared and maintained at 4 °C. Bacteria were cultured overnight in nutrient broth media to reach a mid-log phase around an OD of 0.5. The two bacterial pathogens were coated over the agar plate using the L-shaped glass spreader and then dried. At the same time, 6 mm diameter filter paper disks were dipped in different concentrations of algae nanoparticles (100%, 50%, and 10% *v*/*v* concentration) and placed at specific positions in the agar plates. The agar plates were incubated for 24 h at 37 °C to allow bacterial growth. The diameter of inhibitory zones in mm was measured using a ruler.

### 2.11. Anti-Cancer Cell Proliferation

The MDA-MB-231 breast cancer cells were cultured in Dulbecco’s Modified Eagle’s Medium (DMEM) supplemented with 10% *v*/*v* fetal bovine serum (FBS), 100 μg/mL (100U) penicillin, and 100 μg/mL streptomycin. Incubation was carried out at 37 °C with an atmosphere of 5% CO_2_.

The MTT (3-(4, 5-dimethyl thiazol-2yl)-2, 5-diphenyl tetrazolium bromide) assay was used to assess the anti-cancer activity of algae-coated nanoparticles on MDA-MB-231 breast cancer cells. Tumor cells were plated in 96-well plates at $10^{5}$/well in 100 µL of DMEM. After 24 h, the cells were incubated for 72 h in the presence or in the absence of 10% *v*/*v* Algae-AgNPs. The media were removed, and the cells were incubated with the MTT (5mg/mL) solution for 3 to 4 h for cytotoxicity. After incubation, the MTT solution was removed, and cells were washed three times with 100 µL of DMEM. Then, 100 µL of DMSO (solubilizing reagent) was added to each well and incubated at 37 °C for two hours. The development of a purple color visualized the presence of viable cells due to the formation of formazan crystals. The 96-well plates were read in the spectrophotometer at 590 nm, using DMSO as a blank. Measurements were performed, and the values were plotted as optical density versus the number of living cells.

### 2.12. Statistical Analysis

The results were calculated from the averages of all sample readings and represented as mean ± SD. The treatment of data was performed using Microsoft Excel version 2013 for Windows. Descriptive analysis for a pair-wise comparison of mean values of different variables by the t-test were used with a significant level of *p* ≤ 0.05 and *p* ≤ 0.01.

## 3. Results and Discussion

**Figure 1:**
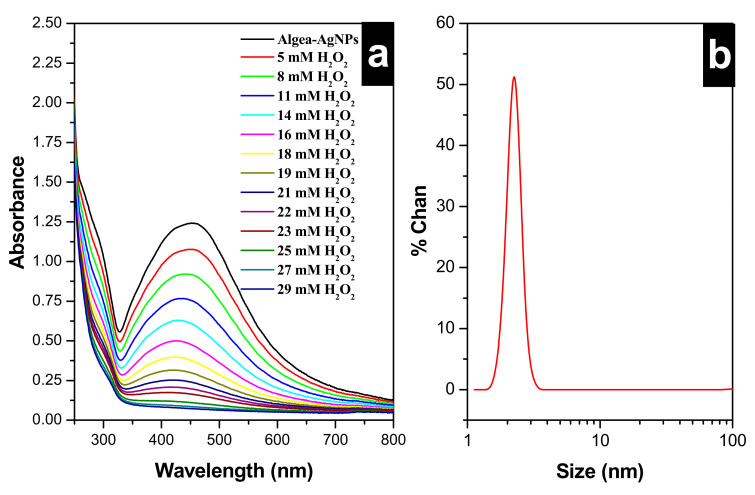
(**a**) UV-Vis responses of Algae-AgNPs solutions upon increasing the concentration of H_2_O_2_ from 5.0 to 29 mM. (**b**) Dynamic light scattering showing the size distribution of the algae-capped silver nanoparticles (Algae-AgNPs).

The UV-vis spectroscopy measurements confirmed the formation of Algae-AgNPs. The maximum Abs at 436 nm was indicative of Ag cupping algae active compounds (Figure 1a). This resonance is originated from the LSPR between the silver and the ligand (Algae active compounds). Dynamic light scattering (DLS) also confirmed the formation of Algae-AgNPs. Figure 1b shows a narrow peak at 2.25 nm corresponding to a 4000 rpm centrifuged Algae-AgNPs. Scanning electron microscopy and elemental mapping (Supplementary Materials Figures S1 and S2) proved the presence of Ag, C, O, and S atoms. Energy-dispersive spectroscopy (EDS) presented in (Supplementary Materials Figure S3) complements the results obtained from SEM single mapping and confirms that the algae active compounds successfully capped the silver nanoparticles. The yield of silver was found to be higher than that of oxygen, carbon, and sulfur. The presence of the three elements (oxygen, carbon, and sulfur) in the EDS spectrum is indicative of the existence of these elements in the structure of the capping shell of the silver. This indicates that silver metal is capped with algae-derived bioactive compounds such as carotenoids, fatty acids, carbohydrates, polyphenols, amino acids, and vitamins [30,44,45]. 

Figure 1 represents the LSPR peak at 436 nm of the Algae-AgNPs, indicating a sufficient degree of dispersion and spherical shape of Algae-AgNPs as well as narrow size distribution. The addition of 0.75 mL of 40.0 mM H_2_O_2_ solution to different volumes of Algae-AgNPs leads to a decrease in the Abs of the surface plasmon resonance of the Ag leading to the bleaching of the Algae-AgNPs’ brownish color to a clear solution.

**Figure 2:**
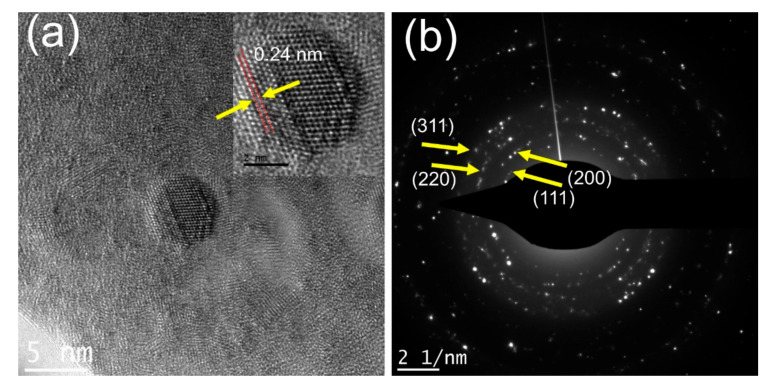
TEM images of Algae-AgNPs prepared by nanoprecipitation of 3:7 *v*/*v* of algae 0.1 M AgNO_3_/plant extract on the holey carbon grid. (**a**) HRTEM image of the Algae-AgNP showing the spherical morphology and crystalline nature of typical 5 nm NP. The inset is showing a magnified view of the NP. (**b**) Selected area electron diffraction (SAED) pattern showing the polycrystalline nature of NP.

Figure 2 represents the structural morphology of the synthesized silver nanoparticles using aberration-corrected high-resolution transmission electron microscopy (HRTEM). Most of the particles were regular spherical shaped (Figure 2a) with the average particle size of 4.5 nm. The results are comparable to those obtained by Heibesh et al. [46]. A typical 4.5 nm Algae-AgNP is shown in Figure 2a. The HRTEM image (in the inset) shows the well-resolved interference fringe patterns separated by 0.24 nm, which corresponded well to the spacing between the (111) plane of fcc (face centered cubic) silver crystal (JCPDS No. 01-087-0597). The selected area electron diffraction (SAED) pattern (Figure 2b) shows the polycrystalline nature of the particles. The patterns were indexed according to (111), (200), (220), and (311) reflections of fcc silver crystal based on their d-spacing of 2.47, 2.13, 1.49, and 1.27 Å.

**Figure 3:**
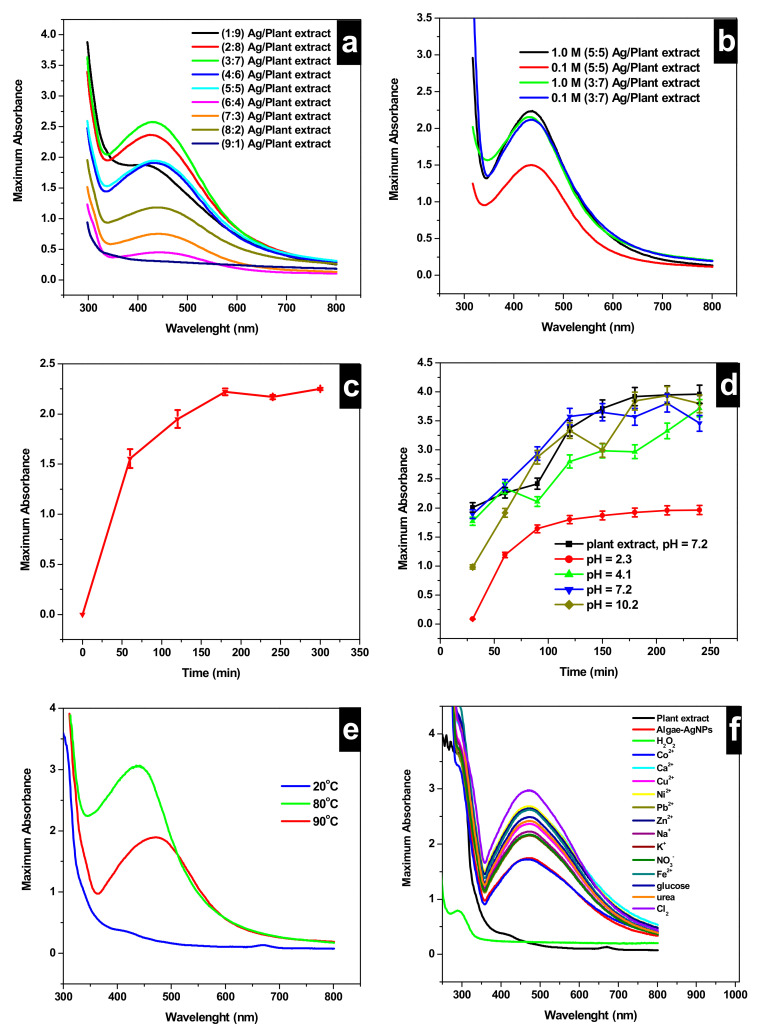
Localized surface plasmon resonance (LSPR) absorbances of Algae-AgNPs; (**a**) as a function of AgNO_3_/plant extract ratios ranging from 1:9 to 9:1 *v*/*v*, (**b**) as a function of AgNO_3_/plant extract ratios and AgNO_3_ molar concentration; (**c**) time effect on the formation of Algae-AgNPs at 80 °C, 0.1 M AgNO_3_, and 3:7 *v*/*v* AgNO3/plant extract ratio, pH_0_ = 7.0; (**d**) pH effect on the formation of Algae-AgNPs at 80 °C, 0.1 M AgNO_3_, and 3:7 *v*/*v* AgNO3/plant extract ratio; equilibrium time varies from 30 to 240 min; (**e**) Temperature effect on the formation of Algae-AgNPs at 25, 80, and 90 °C; 0.1 M AgNO_3_ and 3:7 *v*/*v* AgNO3/plant extract ratio; equilibrium time = 150 min; pH_0_ = 7.0; (**f**) reacted with cation, anion, and neutral species; equilibrium time = 3.0 min; pH_0_ = 7.0, T = 25 °C.

Figure 3 represents the selectivity of Algae-AgNPs toward various neutral, cations, and anion species, and the optimization tests for the synthesis of Algae-AgNPs. First, the ratio of AgNO_3_ to plant extract was optimized (Figure 3a); the ratio of 0.1 M AgNO_3_/Algae extract *v*/*v* was varied between 1:9 and 9:1. An increase in the ratio from 1:9 to 3:7 *v*/*v* yields a peak at 436 nm, indicating the formation of Ag nanoparticles with Abs increased from 1.8 to 2.5 maximum. Upon increasing the ratio from 4:6 to 9:1 *v*/*v*, the maximum Abs of the surface plasmon peak did not improve and was fluctuating between 1.9 and 0.35. Therefore, the ratio of 3:7 *v*/*v* was chosen as the optimum ratio for further investigations. The concentration of AgNO_3_ was also investigated as a parameter that can affect the formation of Ag nanoparticles (Figure 3b). Concentrations of 0.1 M and 1.0 M AgNO_3_ were tested at two different ratios of 5:5 *v*/*v* and 3:7 *v*/*v*. The combinations (5:5 *v*/*v*; 1.0 M), (3:7 *v*/*v*; 1.0 M), and (3:7 *v*/*v*; 0.1 M) (*v*/*v*; AgNO_3_/plant extract, C_AgNO3_) exhibited the highest Abs for LSPR with absorbances of 2.3, 2.2, and 2.1, respectively. The combination (3:7 *v*/*v*; 0.1 M) was used as the optimum combination for further investigations, since less silver nitrate was required.

The optimum time required for Algae-AgNPs formation was investigated using batch experiments, a fixed ratio of (3:7 *v*/*v*; 0.1 M) was equilibrated in an amber bottle under vigorous stirring at 80 °C in a water bath at different timings. Figure 3c represents the absorbances of different aliquots taken at different times. A maximum Abs of 1.75 was attained within the first 50 min, and then the Abs increased to a maximum value of 2.3 on a further increase of the contact time. The process of formation of Algae-AgNPs takes place in two main steps. The first one, where the contact time between the plant extract and the AgNO_3_ solution was less than 50 min, corresponds to the reduction of Ag^+^ to Ag° and the chelating reaction of Ag with the shell formed from bioactive molecules of algae. The second step, which occurs after 50 min, corresponds to an exhausted plant extract that has supplied all active molecules to form a shell around the silver. Therefore, increasing the time does not help improve the Abs of the surface plasmon peak of Algae-AgNPs.

Figure 3d represents the results of the effect of changing the pH on the formation of Algae-AgNPs. Algae-AgNPs were found to be stable in a basic medium. This was concluded from the presence of the LSPR peak of AgNPs at 436 nm. In the three different pH (pH = 7.0, which corresponds to the normal pH of the plant extract, pH = 7.2, and pH = 10.2), the Algae-AgNPs were found to be stable. However, in both adjusted pH values to 7.2 and 10.2, the evolution of the LSPR of Algae-AgNPs did not show smooth curves. They were found to fluctuate between 3.5 and 3.75 Abs values at the timing of 150 min. No trend could be found for pH = 4.1, where the absorbances were found to be fluctuating. In the case of pH = 2.4, a linear increase of the Abs in the first 50 min was observed; then, on a further increase of the contact time to 100 min, a plateau was obtained. This behavior is similar to that obtained during the kinetic studies. However, the absorbances of the LSPR values were not optimum; they were at maximum values of 2.7. Overall, the nanoparticles were not stable at acidic pH, and they are best formed at basic pH specifically at the non-adjusted pH of the plant extract. Therefore, pH = 7.0 was adopted as the optimum pH value of the Algae-AgNPs in further analysis.

The effect of temperature on the formation of Algae-AgNPs was studied at three different temperatures: 25, 80, and 90 °C. Figure 3e showed that no nanoparticles were formed at a room temperature of 25 °C and temperatures below 80 °C. Increasing the temperature to 80 °C leads to the detection of an LSPR peak at 436 nm with a maximum Abs of 3.0. On further increasing the temperature to 90 °C, the LSPR of Algae-AgNPs decreases to 1.9 maximum absorbance. The formation process of Algae-AgNPs can be classified as endothermic between 25 and 80 °C due to the increase of Abs values of LSPR by increasing the temperature. However, it could be classified as exothermic above 80 °C due to the decrease of the LSPR Abs. This behavior could be explained by the competition between the nucleation and growth of nanoparticles that are taking place simultaneously at high temperatures. However, at low temperatures, the growth is favored over nucleation. Similar results were reported by Lui et al. [47] at a high-temperature nucleation rate constant, and the growth rate constant was enhanced. While the nucleation constant was constant between 70 and 80 °C, it rises sharply when the temperature exceeds 80–90 °C, while the growth rate constant was found to increase across the whole temperature range steadily.

Figure 3f shows the UV-vis spectra of Algae-AgNPs reacted with various cations, anions, and neutral species (color change is shown in Supplementary Materials Figure S4). All species show a higher Abs than that of Algae-AgNPs. However, H_2_O_2_ shows the most significant decrease in the Abs with a maximum Abs of 0.70, which is almost closer to the plant extract Abs, which is an indication of the total consumption of the silver nanoparticles catalytic surface by H_2_O_2_. H_2_O_2_ detection using the synthesized Algae-AgNPs was further investigated by optimizing parameters that may influence its exposure, namely, the amount represented by the volume of nanoparticles, time, pH, and temperature effects.

**Figure 4:**
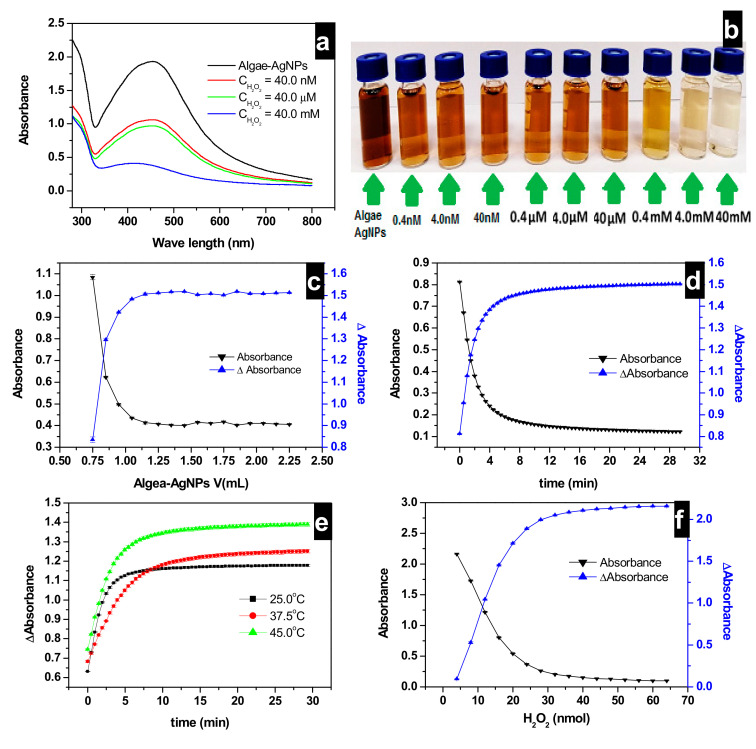
(**a**) LSPR absorbances of Algae-AgNPs at different concentrations of H_2_O_2_; (**b**) color fading of Algae-AgNPs toward H_2_O_2_ concentration increase from 0.4 nM to 40 mM; (**c**) effect of Algea-AgNPs amount on H_2_O_2_ detection at 25 °C, C_i_ = 40 mM; V_i_ (AgNPs) = 0.75 mL; pH_i_ (Algae-AgNPs) = 7.0; (**d**) effect of time on H_2_O_2_ detection by Algae-AgNPs as a function of equilibration time at 25 °C; C_i_ = 40 mM; V (Algae-AgNPs) = 0.75 mL; pHi (Algae-AgNPs) = 7.0; (**e**) effect of temperature on H_2_O_2_ detection by Algae-AgNPs at 25, 37.5, and 45 °C; C_i_ = 40 mM; V (Algae-AgNPs) = 0.75 mL; pHi (Algae-AgNPs) ≈7.0 and (**f**) evolution of LSPR absorbances of the Algae-AgNPs and LSPR ΔAbs against the increase of the concentration of H_2_O_2_ concentration in the 4.7 to 65 nM range.

Figure 4a shows LSPR absorbances of bare Algae-AgNPs and nanoparticles reacted with H_2_O_2_ at concentrations of 40 nm, 40 µM, and 40 mM, respectively. Here, 100 µL of 40 nM H_2_O_2_ added to 0.75 mL of Algae-AgNPs leads to a LSPR Abs decrease from 1.94 to 1.06. A further increase of H_2_O_2_ concentration by adding 100 µL of 40 µM leads to an Abs decrease to 0.97, while the addition of 100 µL of H_2_O_2_ at a concentration of 40 mM almost bleaches out the color of the nanoparticles. A gradual increase of H_2_O_2_ concentration by ten-fold from 0.4 nM to 40 mM (Figure 4b) leads to a gradual brown color fading of the Algae-AgNPs to colorless solution. The effect of Algae-AgNPs volume increase is presented in Figure 4c, which reveals that as the volume of Algae-AgNPs increases from 0.75 to 1.05 mL, the Abs decreased sharply from 1.08 to 0.43. On a further increase of the volume of Algae-AgNPs to 2.5 mL, the amount of H_2_O_2_ detected by Algae-AgNPs was stabilized. This can be due to the complete saturation of the sites of interactions on the Algae-AgNPs surface by H_2_O_2_. 

The decrease in the LSPR Abs with an increase in the volume of Algae-AgNPs can be explained based on the increase of the number of active sites that interact with the H_2_O_2_ when the mass of the material increases. The ΔAbs represented in Figure 4c is obtained by subtracting the sample LSPR Abs from unreacted reference Algae-AgNPs, which was represented with an abrupt increase from 0.83 to 1.48. The plateaus obtained in both curves are attributed to saturated sites of interaction on Algae-AgNPs by H_2_O_2_; an increase in the volume of Algae-AgNPs would lead to increasing the number of sites of interactions. However, H_2_O_2_ has already been trapped in previous active sites. 

**Figure 5:**
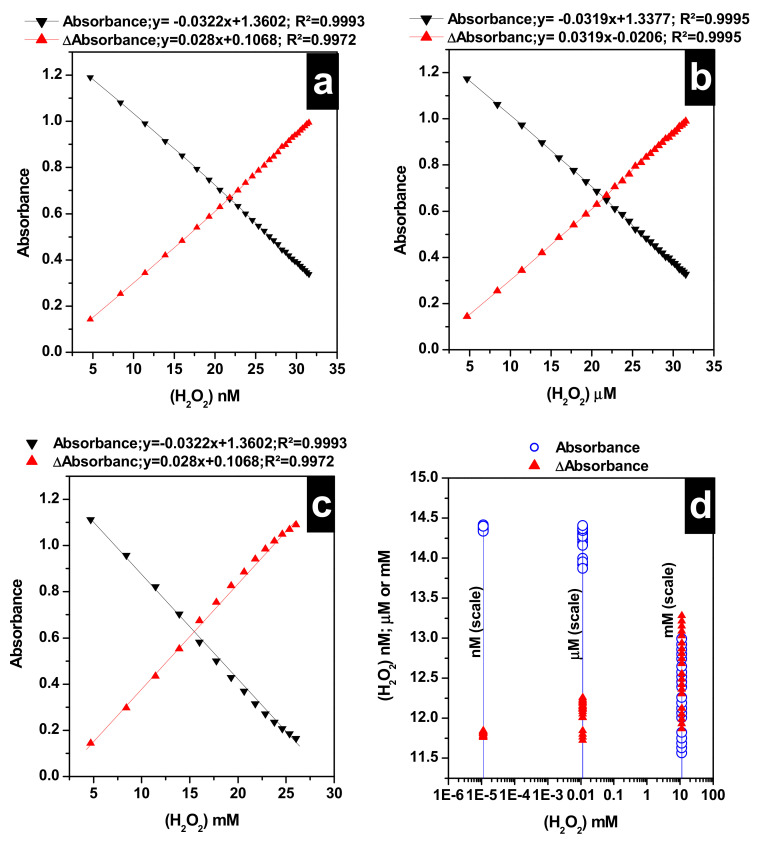
Calibration curves of H_2_O_2_ detection using LSPR absorbances of the Algae-AgNPs and LSPR ΔAbs between the reference and the sample at 25 °C (**a**) 4.7 to 32 nM range; (**b**) 4.7 to 32 µM range; (**c**) 4.7 to 32 mM range; (**d**) determination of unknown concentrations from different ranges, V_unknown_ (H_2_O_2_) = 0.3 mL; V (Algae-AgNPs) = 0.75 mL; pHi (Algae-AgNPs) = 7.0.

Figure 5d represents the absorbances and ΔAbs of the kinetic loop of 0.75 mL of Algae-AgNPs interacted with 0.75 mL of 40 mM H_2_O_2_ solution. A maximum ΔAbs of H_2_O_2_ (1.3) was attained within the first 2.5 min, and then the Abs increases to 1.47 on a further increase of the contact time. The detection process takes place in two main steps. In the first one, the contact time is less than 2.5 min, which corresponds to a rapid interaction. The second process, occurring after 2.5 min, corresponds to an Algae-AgNPs material that is already saturated; therefore, increasing the time does not improve the detection of H_2_O_2_.

The results presented in Figure 4e show the effect of temperature on H_2_O_2_ detection at 25, 37, and 45 °C. The ΔAbs increased with the increasing temperature when faced with a H_2_O_2_ detection decrease. However, the magnitude of such a decrease continues as the temperature is increased to 45 °C. This is because, by increasing the temperature, the attractive forces between the Algae-AgNPs surface and H_2_O_2_ are weakened, and the amount of H_2_O_2_ detected decreases. Most of the H_2_O_2_ detection is a fast process. The detection of H_2_O_2_ occurred in two phases; the first one where detection equilibrium is very fast is at higher concentrations of H_2_O_2_, which becomes relatively constant as the time was increased. Preliminary experiments for the effect of pH on H_2_O_2_ detection by Algae-AgNPs showed that a creamy solution was obtained when changing the pH of Algae-AgNPs to acidic or basic. No LSPR peaks of Algae-AgNPs at 436 nm could be detected. Therefore, it was convenient to study the detection of H_2_O_2_ at the normal pH of the Algae-AgNPs. Figure 4f represents the absorbances and ΔAbs of 0.75 mL of Algae-AgNPs interacted with an increased concentration of H_2_O_2_ solution. The LSPR Abs decreased from 2.64 to 0.5431 when the concentration of H_2_O_2_ increased from 4.0 to 20.0 nM in a linear way; this was faced with an increase in ΔAbs from 0.29 to 1.92. On further increase of the concentration of H_2_O_2_, above 20.0 nM, the LSPR Abs continues to decline but not in a linear way. The curvature of the graph observed above 20.0 nM of H_2_O_2_ is due to the saturation of Algae-AgNPs’ sites of interaction, which leads to a non-linear response. The linear region of response to H_2_O_2_ concentration is presented between the two extremities of the curve. Different experimental situations have shown that the linear range could be extended, and it is dependent on the type of analytes [48].

Next, we assessed the level of detection of H_2_O_2_ in unknown samples prepared by taking 0.3 mL from three H_2_O_2_ solutions, 40 nM, 40 µ M, and 40 mM. Trials to build a single calibration curve that span between nM and mM had failed. Consequently, calibration curves for the Algae-AgNPs test were built in three different ranges: from 4.7 nM to 32 nM, from 4.7 µM to 32 µM, and from 4.7 mM to 32 mM by varying H_2_O_2_ volumes between 0.1 and 2.8 with a step increase of 100 µL in each range. Calibration curves are given in Figure 5a–c for the 4.7–32 nM, 4.7–32 µM, and 4.7–32 mM ranges, respectively. The Algae-AgNPs test exhibited linear responses to H_2_O_2_ in the three ranges for Abs and ΔAbs calibration curves. The limit of detection of the test was determined based on the standard deviation of the response and the slope of the calibration curve in the lowest range according to LOD = 3.3σ/S, where σ is the standard deviation of 10 unknown replicates at 436 nm maximum absorbances. A limit of detection of 1.34 ± 0.02 nM was found for the test when Abs calibration was used, while 1.78 ± 0.02 nM was found when ΔAbs calibration was used instead. The detection limits for both calibration curves were better than those of the H_2_O_2_ sensors reported in Table 1. The quantitation limit of the test was also determined using an LOQ = 10 Lv/S, where Lv is the lowest value recorded by the Algae-AgNPs test, and S represents the slope of the calibration curve in the nM range. 7.31 ± 0.03 nM and 9.67 ± 0.03 nM quantitation limits were obtained when Abs and ΔAbs calibration curves were used, respectively. An ΔAbs calibration curve was presented with a high LOD and LOQ in comparison with the Abs calibration curve. However, the results in Figure 5d showed that values of randomly prepared unknowns of 0.3 mL H_2_O_2_ mixed with 0.75 mL of Algae-AgNPs with theoretical concentrations of 11.41 nM and 11.41 µM were determined accurately using ΔAbs curves with values of 11.81 ± 0.027 nM and 12.08 ± 0.15 µM for the nM and µM ranges, respectively. High values were obtained when Abs curves were used instead. The values obtained were 14.38 ± 0.023 nM and 14.24 ± 0.15 µM for the nM and µM ranges, respectively. No significant difference was found between the Abs and ΔAbs calibration curves in the mM range in determining the concentration of the unknown, showing values of 12.30 ± 0.46 mM and 12.59 ± 0.45 mM, respectively for the unknown in the mM range. This clearly indicates that ΔAbs affords more accurate results when dealing with low concentrations of up to 32 µM. However, for high concentrations, both calibration curves afford identical results.

**Figure 6:**
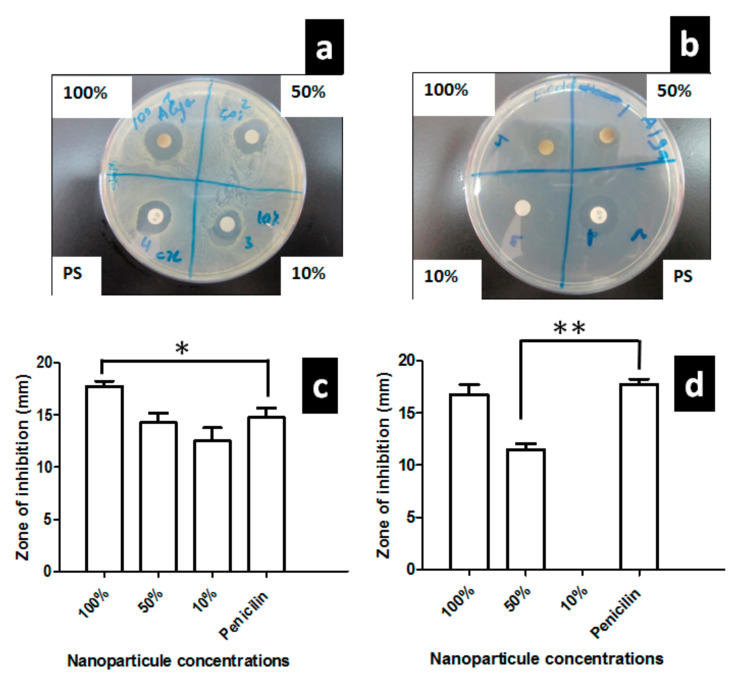
Bacterial growth inhibition using 100%, 50%, and 10% *v*/*v* Algae-AgNPs on *Staphylococcus aureus* (**a**), and *Escherichia coli* (**b**), the average and standard deviation of four replicates of inhibition circles around the inhibited zone of *Staphylococcus aureus* and *Escherichia coli* respectively (**c**,**d**). * *p* ≤ 0.05 and, ** *p* ≤ 0.001.

The antibacterial effect of Algae-AgNPs was tested on two human pathogenic bacteria strains. The results are represented in Figure 6a–d. A significant inhibition of bacterial growth was found for *Staphylococcus aureus* and *Escherichia coli* using 100% and 50% *v*/*v* Algae-AgNPs preparations. However, 10% *v*/*v* was only efficient against *staphylococcus aureus*, but not against *Escherichia coli*. Penicillin/streptomycin as a positive control has the same level of inhibition as 100% solution of Algae-AgNPs on *Escherichia coli*, and only 10% *v*/*v* of Algae-AgNPs solution was able to induce the same level of inhibition as penicillin/streptomycin on *Staphylococcus aureus*. The results showed that Algae-AgNPs were able to stop the bacterial spread and reduce their pathogenicity. The effect was more prominent on *Staphylococcus aureus*, where only 10% *v*/*v* from the original preparation was still efficient to reduce their growth. The synthesized Algae-AgNPs could be used as antiseptic molecules, as previously demonstrated for other Algae-AgNPs [55].

Two main mechanisms are widely used to explain the antibacterial effect of Ag nanoparticles, namely direct association of the nanoparticle with the microorganism’s membrane and charge-mediated mechanism, where the nanoparticle is repelled or attracted to the microorganism membrane by electrostatic forces [56]. Algae-AgNPs showed a zeta potential of 0.200 ± 0.02 mV and negative polarity; this would lead to a strong interaction with *Escherichia coli*, which is known to be high negatively charged compared to *Staphylococcus aureus* [57]. Algae-AgNPs once closer to the microorganism membrane could release high concentration of Ag^+^, which can bind to protein membranes and could result in unstable chemical bonds and deactivation of the proteins [58,59,60]. Transmembrane and peripheric proteins are ion mediators that are involved in ATP generation; in addition, a high concentration of silver in the membrane interface can interact with the oxidative phosphorylation process and limit proton and phosphate permeability [61,62]. Monteiro et al. [63] pointed also to the ability of Ag^+^ as a heavy metal cation to intercalate between purines and pyrimidine bases of DNA, leading to the disruption of the hydrogen bonds between the base pairs, which prevents cell division.

**Figure 7:**
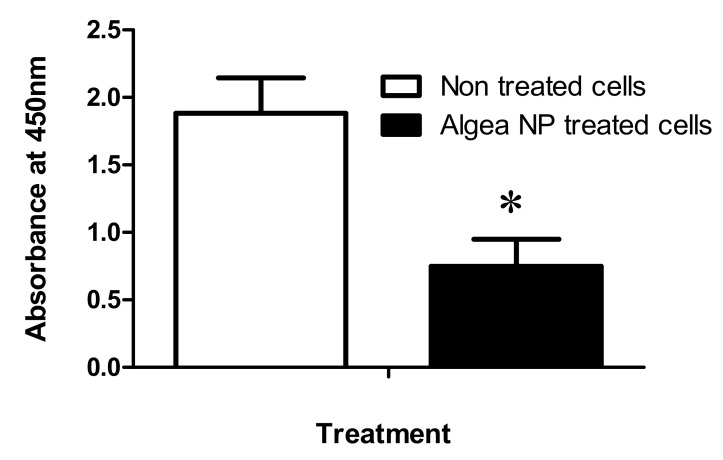
Anti-cancer effect of Algae-AgNPs on MDA-MB-231 breast cancer cell line. Numbers of living cells were determined after treatment with 10% *v*/*v* Algae-AgNPs compared to non-treated cells. A student test was used to determine the difference between treated and non-treated cancer cells (n = 5). * *p* ≤ 0.05.

The MDA-MB-231 human breast adenocarcinoma cell line belongs to a group of aggressive breast cancer. The incubation of MDA-MB-231 with Algae-AgNPs at 10% *v*/*v* dilution for 72 h showed a 50% reduction of tumor cell growth; the results are represented in Figure 7. The cytotoxic effect of Algae-AgNPs is associated with the size and the type of capping agents used for the synthesis of Algae-AgNPs [64]. A smaller particle size penetrates more easily than large particles and causes a cytotoxic effect on tumor cells [65]. The Algae-AgNPs could be used in combination with other conventional anti-cancer drugs such as camptothecin to promote their cytotoxic effect [66].

## 4. Conclusions

Silver nanoparticles (Algae-AgNPs) were prepared from a natural source using a green synthesis approach. The bioactive compounds of the green algae (*Noctiluca scintillans*) were used as a capping shell of the nanoparticles. The plant extract proved to have a self-reducing activity that converted silver ions to metal nanosilver without the need for toxic reducing agents, such as NaBH_4_. The Algae-AgNPs were characterized by various techniques. Optimization of the nanoparticle synthesis was made through controlling the ratio of plant extract to AgNO_3_, pH, time, and temperature. The decomposition of H_2_O_2_ on Algae-AgNPs catalytic surface was found to be pH, temperature, and time dependant. The test was qualified as a colorimetric sensor for H_2_O_2_ detection because of its lowest detection limit for H_2_O_2_ compared to other species. The test showed also a color change from brown to colorless, with H_2_O_2_ presenting the most noticeable change in color. The test assay gave accurate and reproducible values of H_2_O_2_ in unknown samples in three different ranges—nM, μM and mM—using ΔAbs calibration curves. The current study was limited by the inability to build a calibration curve that spans the nM to mM range. However, the smaller detection limit of the assay was lower than reported colorimetric assays in the literature. Thus, this work has highlighted the implications of optimizing the nanoparticles synthesis and parameters affecting H_2_O_2_ detection. Algae-AgNPs were also presented with antibacterial and anti-cancer activities. They were found to have a more prominent effect on *Staphylococcus aureus* when compared with *Escherichia coli* and only at 10% *v*/*v* Algae-AgNPs from the original preparation. Moreover, a 50% reduction of tumor cell growth of MDA-MB-231 human breast adenocarcinoma was assessed using Algae-AgNPs at 10% *v*/*v* dilution for 72 h. These findings make the Algae-AgNPs a suitable assay to detect reactive oxygen species, such as H_2_O_2_, in biological fluids such as urine and blood. Furthermore, it could be proposed as a therapeutic agent against bacterial infection and cancer disease.

## Figures and Tables

**Table 1:** Comparison of analytical performances of different H_2_O_2_ colorimetric sensors.

Method	Limit of Detection (LOD)	Linear Range	Reference
SAgNPs	3.70 µM	0.45–121 µM	[15]
	10.5 nM	4.70–32.0 nM	
TMB + H_2_O_2_ + I^−^ reaction	1.1 μM	1.7–166.7 μM	[49]
AgNPs/GQDs	30 μM	0.5–8 mM	[50]
Brown Algae AgNPs	8.6 nM	1–120 μM	[51]
(Fe_3_(PO_4_)_2_·8H_2_O NFs)	5.0 nM	1 × 10^−5^–2.5 mM	[52]
Ni-MOF nanosheet	8.0 nM	0.04–160 μM	[53]
Cellulose AgNPs	112 µM	60–600 µM	[43]
Pt-Cu nanoparticles	0.17 μM	0.50 μM–22.3 mM	[54]
Colorimetric sensor Algae-AgNPs	Abs 1.34 ± 0.02 nM	4.70–32.0 nM	This work
	ΔAbs 1.78 ± 0.02 nM	4.70–32.0 nM	

## Supplementary Materials

The following are available online at https://www.mdpi.com/2079-4991/10/9/1861/s1, Figure S1: Scanning electron microscopy and Figure S2: Elemental mapping (single layer) of Ag, O, C, S and N of Algae-AgNPs prepared by nanoprecipitation of 7:3 *v*/*v* of algae extract/0.1 M AgNO_3_, Figure S3: Energy-dispersive spectroscopy (EDS) spectra and atomic ratio of corresponding elements in the Algae-AgNPs, Figure S4: Photographs of Algae-AgNPs exposed to different interfering species with H_2_O_2_, showing H_2_O_2_ with the only noticeable change in color. Initial concentration of 10.0 nM, time of equilibrium 40 min.
