# Supplementary material for: Green Synthesis, Characterization, Antimicrobial, Anti-Cancer, and Optimization of Colorimetric Sensing of Hydrogen Peroxide of Algae Extract Capped Silver Nanoparticles
Source: Nanomaterials (Basel). 2020 Sep 17;10(9):1861. doi: 10.3390/nano10091861 (PMC7557820; doi:10.3390/nano10091861)
Supplement: Supplementary file 1 [file nanomaterials-10-01861-s001.pdf]

Supporting Information

Green synthesis, characterization, and optimization of colorimetric sensing of hydrogen peroxide of algae (*Noctiluca scintillans*) extract capped silver nanoparticles.

Abdelaziz Elgamouz*[1], Hamid Idriss[1], Chahlaa Nassab[1], Alaa Bihi[1], Khalid Bajou[2], Kamrul Hasan[1], Mohammad Abu Haija[3], Shashikant P. Patole[4].

[1]Department of Chemistry, College of Sciences, PO. Box 27272, Sharjah, University of Sharjah, United Arab Emirates.

[2]Department of Biology, College of Sciences, PO. Box 27272, Sharjah, University of Sharjah, United Arab Emirates.

[3]Department of Chemistry, Khalifa University of Science and Technology, Abu Dhabi, PO. Box 127788, Abu Dhabi, United Arab Emirates.

[4]Department of Physics, Khalifa University of Science and Technology, Abu Dhabi, PO. Box 127788, Abu Dhabi, United Arab Emirates

* Correspondence: aelgamouz@sharjah.ac.ae; Tel.: +971 65166 769, E-mail: aelgamouz@sharjah.ac.ae.

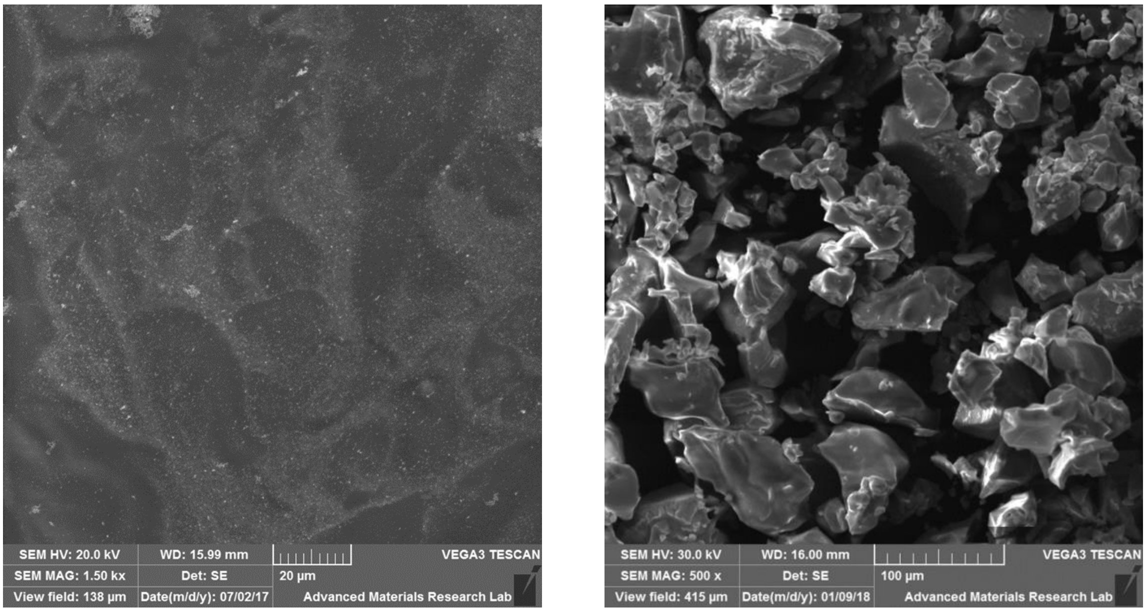

Figure. S1 Micrograph of Algae-AgNPs (a) SEM MAG = 1.50 Kx (b) SEM MAG = 500 Kx prepared by nanoprecipitation of 7:3 v/v of algae extract/0.1 M $AgNO_3$.

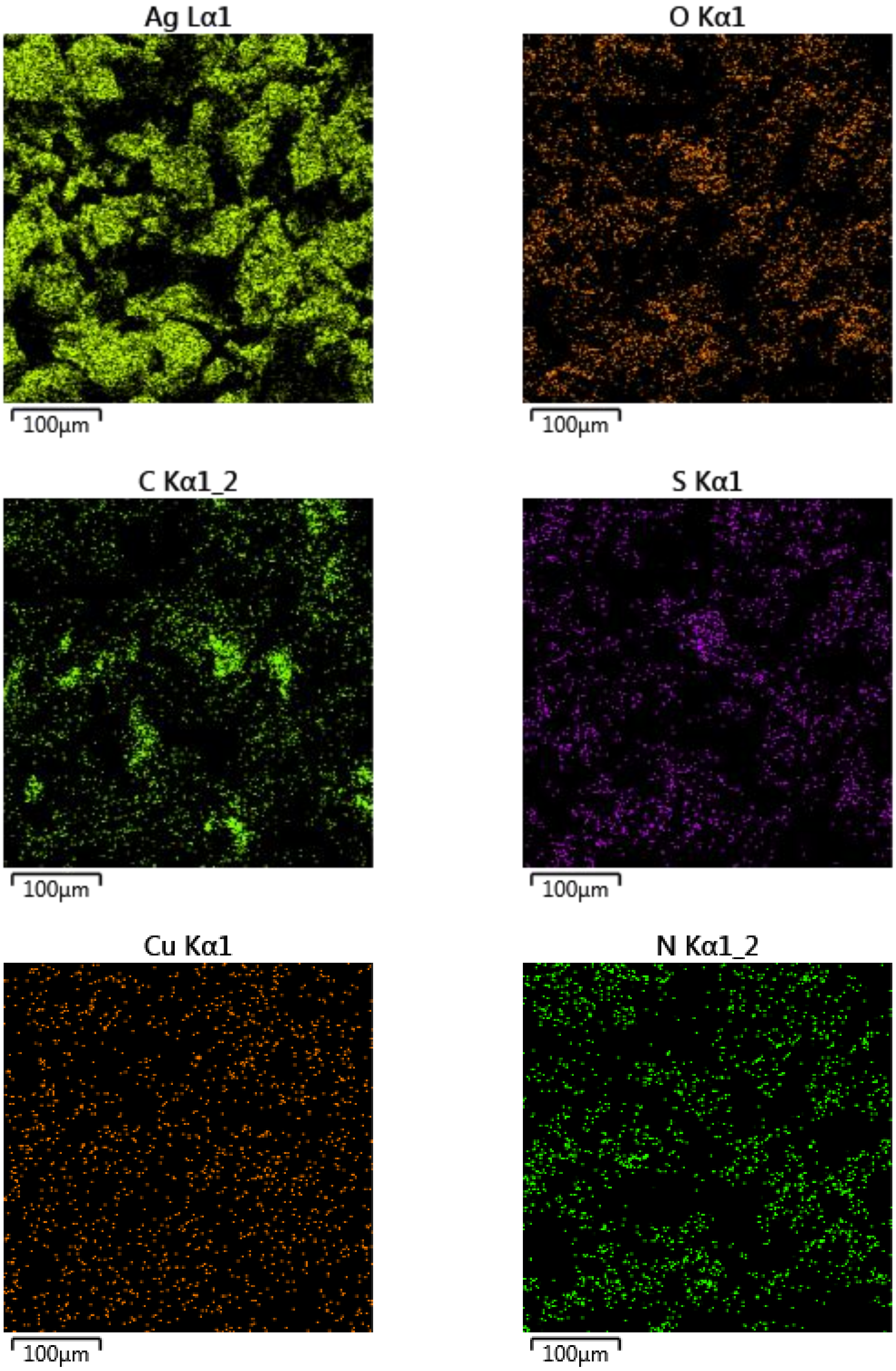

Figure. S2 Scanning electron microscopy elemental mapping (single layer) of Ag, O, C, S and N of Algae-AgNPs prepared by nanoprecipitation of 7:3 v/v of algae extract/0.1 M $AgNO_3$.

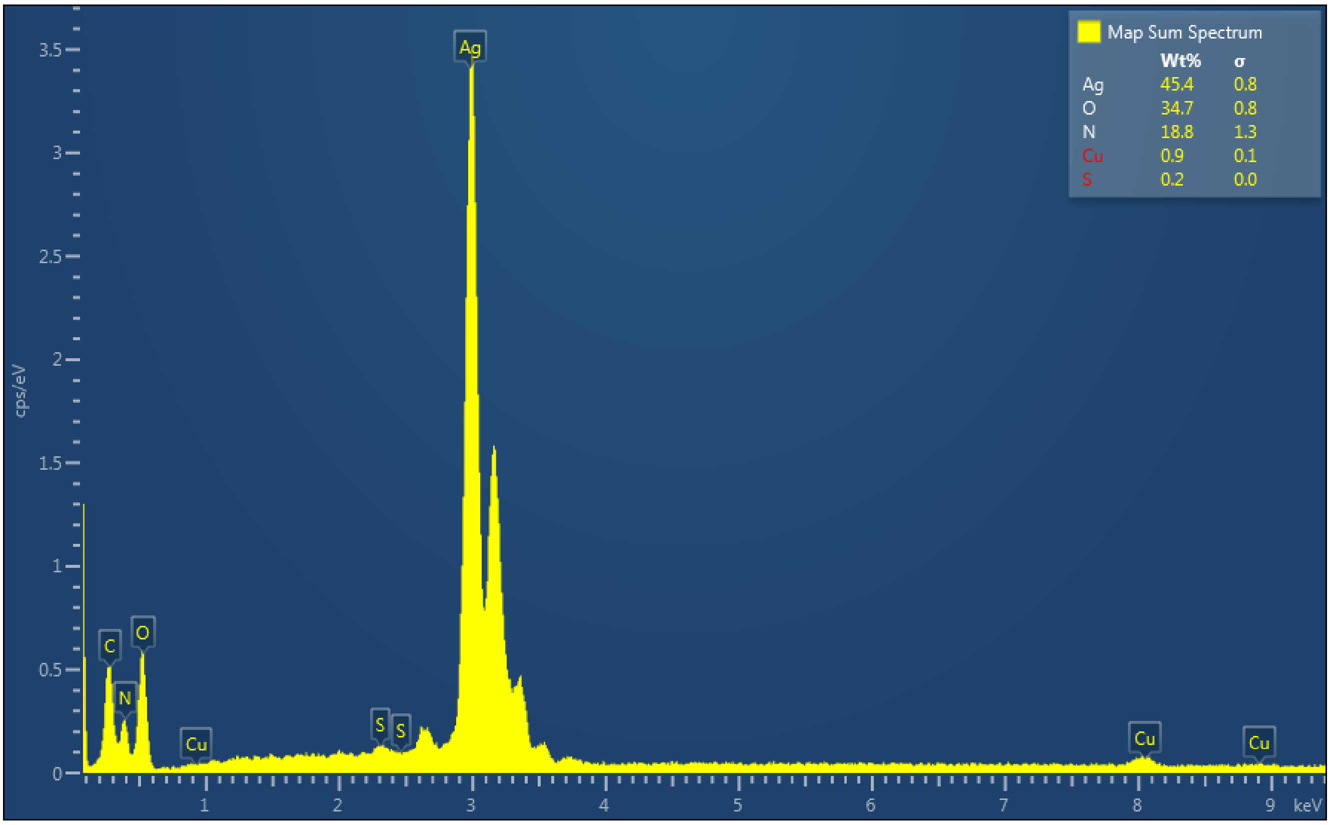

Figure. S3 Energy disperse spectroscopy (EDS) spectra and atomic ratio of corresponding elements in the Algae-AgNPs.

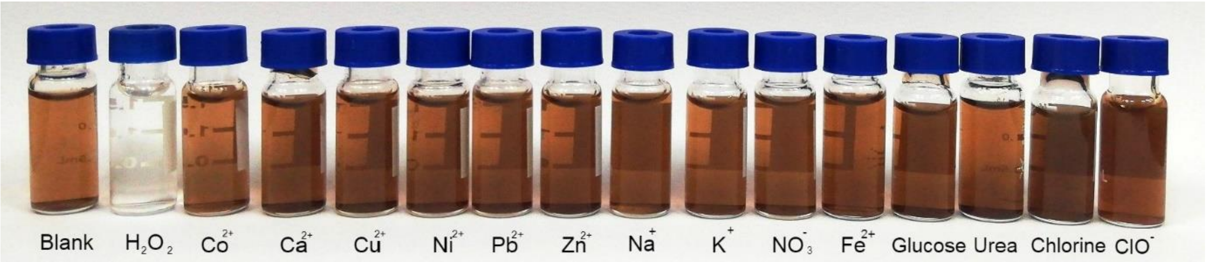

Figure. S4 Photographs of Algae-AgNPs exposed to different interfering species with H_2O_2, showing H_2O_2 with the only noticeable change in colour. Initial concentration of 10.0 nM, time of equilibrium 40 min.
